# Cognitive Flexibility Predicts PTSD Symptoms: Observational and Interventional Studies

**DOI:** 10.3389/fpsyt.2018.00477

**Published:** 2018-10-04

**Authors:** Ziv Ben-Zion, Naomi B. Fine, Nimrod Jackob Keynan, Roee Admon, Nili Green, Mor Halevi, Greg A. Fonzo, Michal Achituv, Ofer Merin, Haggai Sharon, Pinchas Halpern, Israel Liberzon, Amit Etkin, Talma Hendler, Arieh Y. Shalev

**Affiliations:** ^1^Sagol Brain Institute Tel-Aviv, Wohl Institute for Advanced Imaging, Tel Aviv Sourasky Medical Center, Tel-Aviv, Israel; ^2^Sagol School of Neuroscience, Tel-Aviv University, Tel-Aviv, Israel; ^3^Psychological Trauma Care Center, Shaare-Zedek Medical Center, Jerusalem, Israel; ^4^School of Psychological Sciences, Faculty of Social Sciences, Tel-Aviv University, Tel-Aviv, Israel; ^5^Department of Psychology, University of Haifa, Haifa, Israel; ^6^Department of Psychiatry and Behavioral Sciences, Stanford University, Stanford, CA, United States; ^7^Stanford Neurosciences Institute, Stanford University, Stanford, CA, United States; ^8^Veterans Affairs Palo Alto Healthcare System, The Sierra Pacific Mental Illness, Research, Education, and Clinical Center (MIRECC), Palo Alto, CA, United States; ^9^Trauma Unit and Department of Cardiothoracic Surgery, Shaare-Zedek Medical Center, Jerusalem, Israel; ^10^Faculty of Medicine, Hebrew University of Jerusalem, Jerusalem, Israel; ^11^Sackler Faculty of Medicine, Tel-Aviv University, Tel-Aviv, Israel; ^12^Department of Anesthesiology and Critical Care Medicine, Institute of Pain Medicine, Tel Aviv Sourasky Medical Center, Tel-Aviv, Israel; ^13^Pain Management and Neuromodulation Centre, Guy's & St Thomas' NHS Foundation Trust, London, United Kingdom; ^14^Department of Emergency Medicine, Tel Aviv Sourasky Medical Center, Tel-Aviv, Israel; ^15^Department of Psychiatry, University of Michigan, Ann Arbor, MI, United States; ^16^Department of Psychiatry, NYU Langone Medical Center, New York, NY, United States

**Keywords:** Post-Traumatic Stress Disorder (PTSD), neurocognitive functioning, cognitive flexibility, resilience factors, risk factors, cognitive training intervention

## Abstract

**Introduction:** Post-Traumatic Stress Disorder (PTSD) is a prevalent, severe and tenacious psychopathological consequence of traumatic events. Neurobehavioral mechanisms underlying PTSD pathogenesis have been identified, and may serve as risk-resilience factors during the early aftermath of trauma exposure. Longitudinally documenting the neurobehavioral dimensions of early responses to trauma may help characterize survivors at risk and inform mechanism-based interventions. We present two independent longitudinal studies that repeatedly probed clinical symptoms and neurocognitive domains in recent trauma survivors. We hypothesized that better neurocognitive functioning shortly after trauma will be associated with less severe PTSD symptoms a year later, and that an early neurocognitive intervention will improve cognitive functioning and reduce PTSD symptoms.

**Methods:** Participants in both studies were adult survivors of traumatic events admitted to two general hospitals’ emergency departments (EDs) in Israel. The studies used identical clinical and neurocognitive tools, which included assessment of PTSD symptoms and diagnosis, and a battery of neurocognitive tests. The first study evaluated 181 trauma-exposed individuals one-, six-, and 14 months following trauma exposure. The second study evaluated 97 trauma survivors 1 month after trauma exposure, randomly allocated to 30 days of web-based neurocognitive intervention (*n* = 50) or control tasks (*n* = 47), and re-evaluated all subjects three- and 6 months after trauma exposure.

**Results:** In the first study, individuals with better cognitive flexibility at 1 month post-trauma showed significantly less severe PTSD symptoms after 13 months (*p* = *0.002*). In the second study, the neurocognitive training group showed more improvement in cognitive flexibility post-intervention (*p* = *0.019*), and lower PTSD symptoms 6 months post-trauma (*p* = *0.017*), compared with controls. Intervention- induced improvement in cognitive flexibility positively correlated with clinical improvement (*p* = *0.002*).

**Discussion:** Cognitive flexibility, shortly after trauma exposure, emerged as a significant predictor of PTSD symptom severity. It was also ameliorated by a neurocognitive intervention and associated with a better treatment outcome. These findings support further research into the implementation of mechanism-driven neurocognitive preventive interventions for PTSD.

## Introduction

Post-Traumatic Stress Disorder (PTSD) is a severe mental disorder with profound public health impact due to its high prevalence, persistence, and associated functional impairment (1, 2). PTSD symptoms are commonly observed shortly after trauma exposure and their initial severity has been associated with a high risk of non-recovery (3–7). Longitudinal studies of stress exposure have documented significant heterogeneity in symptoms trajectories (i.e., PTSD symptoms in humans; freezing as avoidance in animals), suggesting a heterogeneity of underlying neurobiological mechanisms (3, 8–11).

Neurocognitive deficits linked with the emergence of PTSD (12, 13) concern working memory, information processing speed and verbal learning, and short-term and declarative memory (14, 15), attention, and executive functioning (16, 17). PTSD has been repeatedly associated with difficulties in response inhibition, attentional switching and flexibility (18–22), and these features were hypothetically linked with PTSD patients' difficulties to disengage attention from a salient stimuli (23). Neuroimaging studies of PTSD have, similarly, documented altered prefrontal network activity in tasks requiring inhibition and attentional switching [e.g., (24, 25)]. These neurocognitive targets may serve as risk-resilience factors for the development and/or maintenance of post traumatic symptoms. Evidence has also shown that better neurocognitive functions were associated with to lower rates of PTSD diagnosis (26).

Previous work suggests that the central nervous system's activity may be altered by experience at multiple levels of neural organization (27–29). These and other findings suggest that early aftermath of traumatic events might be a stage of increased brain plasticity and therefore a period of accelerated learning (30, 31). Furthermore, evidence suggests that targeted, intensive, repetitive and adaptive task engagement can powerfully shape neural organization and function (32), and as such it provides an exceptional opportunity to investigate neurobehavioral modifications and their implications.

To date, preventive interventions for PTSD have neither considered nor specifically targeted survivors' neurocognitive capabilities and did not evaluate PTSD clinical and neurocognitive dimensions over time. Longitudinally exploring the latter may enhance our understanding of disease progression and prevention, and provide important information on survivors' susceptibility to develop PTSD. Observing the temporal sequence of clinical and neurocognitive changes, shortly after trauma exposure, may, additionally lead to devising new and better-targeted preventive interventions.

This work longitudinally examines the association between clinical symptoms and neurocognitive functions in recent trauma survivors, and the contribution of specific neurocognitive functions to PTSD pathogenesis. In a first study we explored the relationship between neurocognitive functions recorded 1 month after trauma exposure and PTSD symptoms at different time intervals from trauma exposure (“Study 1”). In a second study, we evaluated the association between neurocognitive functions at 1 month and the effect of an early neurocognitive interventions (“Study 2”). Our main hypothesis was that better neurocognitive functioning shortly after trauma will be associated with less severe PTSD symptoms 13 months later (“Study 1”). Our auxiliary hypothesis was that an early neurocognitive intervention will improve cognitive functioning and reduce PTSD symptoms (“Study 2”).

## Methods

### Participants

Participants were adult survivors of traumatic events, admitted to two general hospital in Israel for treatment of traumatic injury. In both studies participants were considered for a telephone screening interview if they met the following inclusion criteria: (i) Age 18–65 years (ii) Able to read and comprehend Hebrew or English (language used in neurocognitive tasks) (iii) Arrived in the ER because of one of the following: car accidents, terrorist attacks, work accidents, home accidents, burns, physical assault, large-scale disaster. To reduce confounds related to concurrent disorders, the studies' exclusion criteria included: (i) survivors with open head injury or in a coma upon ER arrival; (ii) survivors with known medical condition that interfere with their ability to give informed consent, cooperate with screening and/or treatment; (iii) survivors with chronic PTSD from previous events, and those with current or lifetime psychotic illness or current substance abuse, suicidal risk or mental disorders or conditions that constitute treatment priority; (iv) individuals using psychotropic medication or recreational drugs in the week that precedes the assessment. In addition, survivors currently treated with benzodiazepines or those receiving cognitive behavioral therapy for their posttraumatic symptoms were excluded.

### Clinical instruments

In both Study 1 & Study 2, we used the following measurements:

#### The clinician-administered PTSD scale (CAPS)

Structured interview for assessing posttraumatic stress disorder (PTSD) diagnostic status and symptom severity. We used a version of the CAPS that combines DSM-IV and DSM-5 criteria in order to keep continuity. The CAPS contains explicit, behaviorally anchored probes for each PTSD symptom criteria. The CAPS symptom severity scores were obtained by summing all individual items. The Hebrew version used in this work was cross-translated and compared with the original English instruments. Internal consistency of CAPS-5 was 0.88 and test-retest reliability was 0.78 (33).

#### The structured clinical interview for DSM-IV (SCID)

Structured clinical interview evaluating current and lifetime (pre-event) Axis I mental disorders (34).

### Neurocognitive functions measurement

In both Study 1 & Study 2, we used the following measurements:

#### Webneuro

An Internet-based, comprehensive battery of neurocognitive functioning, previously validated against traditional neurocognitive tests (35). To reduce the effect of learning between testing sessions, we used two WebNeuro versions that included the use of different stimuli and trial sequences. WebNeuro accommodates both Hebrew and English languages. To standardize testing conditions, all tests were taken in our laboratory in the receiving hospital rather than participants' homes. Performance in the different tasks were calculated using an automated software program, which derived standardized Z-scores for each participant at each of the following 11 neurocognitive composite domains: motor coordination, processing speed, sustained attention, controlled attention, cognitive flexibility, response inhibition, working memory, recall memory, executive function, emotion identification, and emotional bias.

The WebNeuro battery included the following main tasks:

1. **Digit Span (working memory):** Participants indicated whether the current letter on the screen matches a letter presented N-steps back. Successful performance on this task required constant updating of memory storage and focus.
2. **Memory Recognition Task (recall memory**): On each trial, a list of 20 words was presented. In part 1, participants were presented with 20 sets of three response words and were instructed to select the one word that was previously presented. In part 2, which was completed 10 min later, participants were again presented with 20 sets of three response words, from which they had to select one word from each set that they believed that was previously presented. Performance is measured on total immediate recall and delayed recall response.
3. **Stroop Task (response inhibition)** (36): Participants were presented with color names printed in either matched or mismatched colors (e.g., the word RED printed red or in green ink). Their task was to indicate the ink color, while disregarding the meaning of the word. Successful performance on this task required participants to inhibit the reaction to the word and prioritize the reaction to the ink color.
4. **Maze Task (executive function):** Participants were instructed to memorize a complicated sequence of flashing dots, and then to re-enact it three times in a row without errors. Successful performance on this task required storing information in working memory, resisting impulsive moves when re-enacting the sequence, and responding as fast as possible.
5. **Emotional Identification Task:** In this task, a series of faces was presented on the screen, each displaying one of six emotional expressions (fear, anger, disgust, sadness, happiness, or neutral). On each trial, participants select as quickly as possible the emotion label that best matches each face from six response buttons displayed beneath the face (“fear,” “anger,” “disgust,” “sad,” “happy,” “neutral”). Accuracy and RT for each emotion were measured.
6. **Emotional Bias Task:** In this task, sets of two faces were presented. In each set, one face is repeated from the previous Emotion Identification task, and one face is new. Participants use the mouse to select which of the two faces they remember from the previous task. Response time for each emotion is measured.
7. **Go/No-Go Task (response inhibition):** This classic task required maintaining balance between automatic responding (impulsivity) and response suppression (inhibition) to a stimulus presented.
8. **Motor tapping Task (motor coordination):** Participants were required to tap a circle on the touch-screen with their index finger, as fast as possible for 60 s. The dependent variable was total number of taps with the dominant hand and pauses between taps.
9. **Switching of Attention Task (cognitive flexibility):** This task is a computerized adaption of the manual Trail Making test (37). Each participant was presented with a mixture of 13 numbers and 12 letters, and was instructed to switch back and forth between numbers and letters in an ascending pattern (e.g., 1-A-2-B, etc.). Successful performance on this task required continuous shifting between task sets, while keeping in mind the previously connected items. Response accuracy, completion time and average connection time were measured.

### Procedure

In both studies, a member of the research team identified potentially trauma-exposed patients using the ER medical records. Within 7–14 days after trauma exposure, and after being discharged from the hospital, the identified individuals were contacted by telephone for an initial screening. The telephone screening was conducted by MA level clinicians that were trained in the specific assessment tools [see (38) for detailed description]. After verbal consent, the PTSD Checklist (PCL) was administered to assess risk of PTSD development. Those who met PTSD symptom criteria (except the 1 month duration) and did not meet any of the exclusion criteria, received verbal information about the research and were invited to a clinical assessment session within 1 month post-trauma. The clinical assessment included 2-h structured clinical interviews (CAPS, SCID) and 1-h neurocognitive evaluation (WebNeuro). Participants received financial remuneration at the end of the assessment, according to the ethics committee regulations and approval. In both studies, two follow-up clinical and neurocognitive assessments were conducted; Study 1 assessments were conducted at 1, 6, and 14 months after trauma (TP1, TP2 and TP3 accordingly), whereas Study 2 assessments were conducted at 1, 3, and 6 months after trauma (T1, T2, and T3 accordingly).

Study 1 did not include any intervention or treatment, while Study 2 included a neurocognitive intervention of a daily 30 min sessions for 30 days. The intervention details in Study 2 are fully described in Fine et al. (38) and are only summarized here. Participants were blindly allocated to either a neurobehavioral training group or one of two control groups. The training group included classic paradigm tasks that specifically targeted executive function (e.g., working memory, task switching, resisting interference) and emotional reactivity and regulation. The first control group was engaged in web-based tasks with similar visual appeal that do not address specific neurobehavioral domains such as card games, Tetris, obstacle course, classic computer games (e.g., Pac-Man), visual search tasks, and different kinds of matching tasks. These tasks mainly train dexterity (such as clicking quickly with the computer mouse), however we can't fully ensure that they did not improve any neurocognitive domain (e.g., executive functions). The second control group consisted of visually appealing reading tasks whose contents were limited to emotionally neutral topics (e.g., nature, geography). Intervention included a combined regimen of “Lumosity” neurocognitive training games and “MyBrainSolutions” emotional bias training. On each training day, the active group were given eight tasks, chosen at a random sequence within each category (categories were: focus/inhibition, working memory, task shifting, emotion recognition/resisting distraction, positivity bias) and in the control groups they chose eight out of ten control tasks. All tasks were designed to be dynamic, adaptive, and continually engaging, such that they increase in difficulty level as performance improves

In Study 1, 2,944 trauma patients underwent a telephone screening interview. Of those, *n* = 525 (18%) had acute stress disorder (ASD) symptoms, and 350 (12%) were invited for clinical assessments. A total of 181 (6%) individuals were enrolled to the study. At the point of writing this work, 97 participants completed the second, 6 months' assessment (TP2), and 61 completed the third, 14 months' assessment (TP3).

In Study 2, 3,387 trauma patients underwent a telephone screening interview. Of those, *n* = 643 (19%) had ASD symptoms, and 347 (10%) were invited for clinical assessments. A total of 111 (3%) individuals were enrolled to the study, out of which *n* = 14 were assigned as a follow-up group without intervention, for technical reasons. The 97 remained participants were randomized into three groups: (1) Neurocognitive training (*n* = 50); (2) Game tasks (*n* = 30); (3) Reading tasks (*n* = 17). 86 participants completed the second, 3 months' assessment (T2), and 78 completed the third, 6 months' assessment (T3).

### Data analysis

IBM SPSS Statistics for windows, Version 23.0, was used for the statistical procedures. For each separate analysis, participants with extreme scores greater than 2.5 SD from the mean (in absolute values) in the relevant variables were defined as outliers, and hence were excluded from the analysis. Pearson correlations coefficients and their significance were computed between neurocognitive Z-scores (main predictors) and CAPS total scores (main outcome measure). Independent *t*-tests compared between-groups effects, for examining changes in both cognitive flexibility and PTSD symptom severity post-intervention. Effect sizes were reported using Cohen's *d* for the conducted *t*-tests. All statistical tests used α of 0.05 with one-sided a-priori hypothesis. Bonferroni correction was used when necessary to counteract the problem of multiple comparisons. For Study 2, in each group (treatment or control), we excluded participants which completed less than 60% of the practices (i.e., dropouts), under the assumption that neurocognitive modification requires repeated and extensive training “dose.”

## Results

### Demographic characteristics

Study 1 included 181 participants at TP1 (Age = 34.59±11.80, 97 Females), 97 at TP2 (Age = 35.38 ± 12.20) and 61 at TP3 (Age = 35.46 ± 12.67, 31 Females). Study 2 included 97 participants at T1 (Age = 36.42±11.41, 53 Females), 86 at T2 (Age = 37.38 ± 12.49, 48 Females) and 78 at T3 (Age = 38.38±12.79, 44 Females). In each one of the studies, no significant differences in age or gender were found between the three time points (*p* > 0.05 for all).

In Study 2, the active group consisted of 50 participants (Age = 35.08 ± 10.13, 26 Females), and the control group (both games and reading) consisted of 47 participants (Age = 37.85 ± 12.58, 27 Females). No significant differences were found between the two groups in age [*t*_(95)_ = −*1.198, p* = 0.234], gender [$\chi_{\left(1\right)}^{2}$ = 0.290*, p* = 0.590], or initial symptom severity at T1 (CAPS-5: *p* = 0.976; CAPS-4: *p* = 0.919) and T2 (CAPS-5: *p* = 0.545; CAPS-4: *p* = 0. 868). To combine the two control arms (reading vs. games) we tested that no differences were present between them in age (*p* = 0.991), gender (*p* = 0.905) and initial symptom severity (CAPS-5: *p* = 0.374; CAPS-4: *p* = 0.826), hence conjoined to one control arm. No significant differences were found between the 52 participants who completed at least 60% of the practices (i.e., completers) and the 45 participants who did not (i.e., dropouts), on age (*p* = 0.818), gender (*p* = 0.142) or initial symptom severity (CAPS-5: *p* = 0.486; CAPS-4: *p* = 0.250).

### Early cognitive flexibility predicts subsequent PTSD symptoms

To test our main hypothesis that better general neurocognitive functions at 1 month after trauma will predict less severe PTSD symptoms 14 months post trauma exposure (Study 1), Pearson correlations were calculated between all 11 neurocognitive domains Z-scores at TP1 and PTSD symptom severity (CAPS-4 and CAPS-5 total scores) at TP3 (see Table 1).

**Table 1:** Pearson correlations between Study 1 participants' neurocognitive domains Z-scores at 1 month after trauma (TP1) and PTSD symptom severity at 14 months after trauma (TP3).

**TP1 Neurocognitive Domains Z-Scores**	**Number of Participants (*n*)**	**Correlation with CAPS-4 Total Scores (*r*)**	**Correlation with CAPS-5 Total Scores (*r*)**
Motor Coordination	44	−0.196	−0.221
Controlled Attention	54	−0.067	−0.139
Sustained Attention	53	−0.217	−0.269^*^
Emotional Bias	54	−0.062	−0.136
Cognitive Flexibility	54	−0.389^**^	−0.394^**^
Response Inhibition	54	0.021	−0.027
Identifying Emotions	54	−0.085	−0.115
Processing Speed	52	−0.120	−0.215
Recall Memory	54	−0.175	−0.185
Working Memory	54	−0.210	−0.243^*^
Executive Function	54	−0.094	−0.079

* p < 0.05 one-sided;

** *p < 0.004 one-sided*.

**Figure 1:**
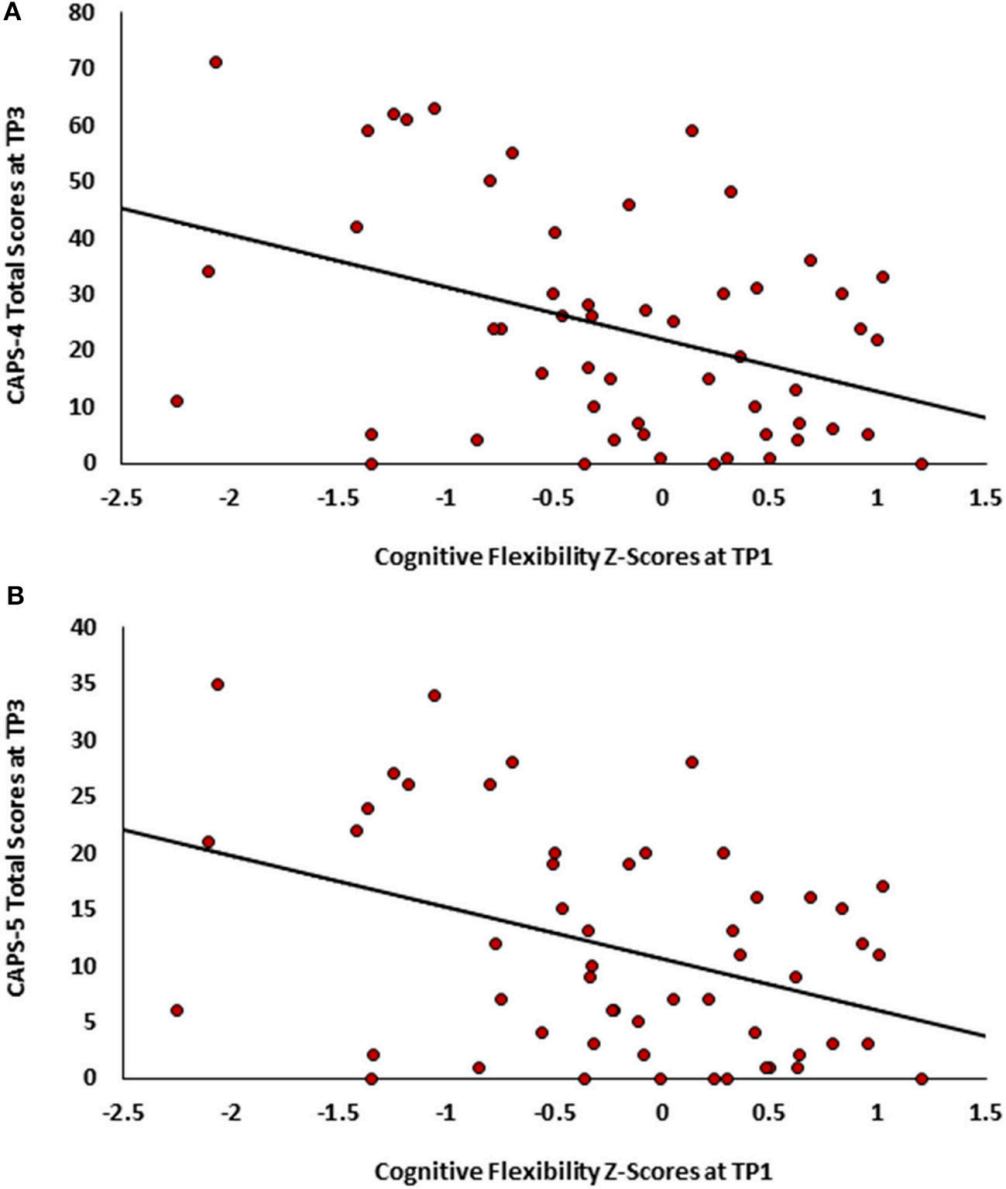
Scatter plots depicting PTSD symptoms severity at TP3 as a function of cognitive flexibility at TPI. **(A)**- CAPS-4 total scores. **(B)** CAPS-5 total scores.

To test our main hypothesis that better general neurocognitive functions at 1 month after trauma will predict less severe PTSD symptoms 14 months post trauma exposure (Study 1), pearson correlations were calculated between all TP1 neurocognitive domains and TP3 PTSD symptom severity (CAPS-4/5 total scores). After bonferroni correction, results revealed a single significant correlation in cognitive flexibility domain (see Table 1), such that higher cognitive flexibility was associated with lower future symptoms (see Figure 1). Controlling for participants' age, gender, marital status, type of trauma and initial symptom severity, this correlation remained statistically significant (CAPS-5: *r* = −0.292, *p* = 0.036; CAPS-4: *r* = −0.274, *p* = 0.046). The association between flexibility and PTSD symptoms at earlier time-points was not significant among 126 TP1 participants (CAPS-5: *r* = −0.061, *p* = 0.248; CAPS-4: *r* = −0.051, *p* = 0.284), and marginally significant among 82 TP2 participants (CAPS-5: *r* = −0.141, *p* = 0.098; CAPS-4: *r* = −0.177, *p* = 0.056).

In study 2, among the follow-up group, pearson correlations were calculated between T1 cognitive flexibility and PTSD symptom severity (CAPS-5 total scores) at all time-points. Results revealed non-significant correlations at T1 (*n* = 14, *r* = −0.013, *p* = 0.482) and T2 (*n* = 10, *r* = 0.049, *p* = 0.447), but a significant negative correlation at T3 (*n* = 10, *r* = −0.558, *p* = 0.047).

### Early treatment improves cognitive flexibility and subsequent PTSD symptoms

**Figure 2:**
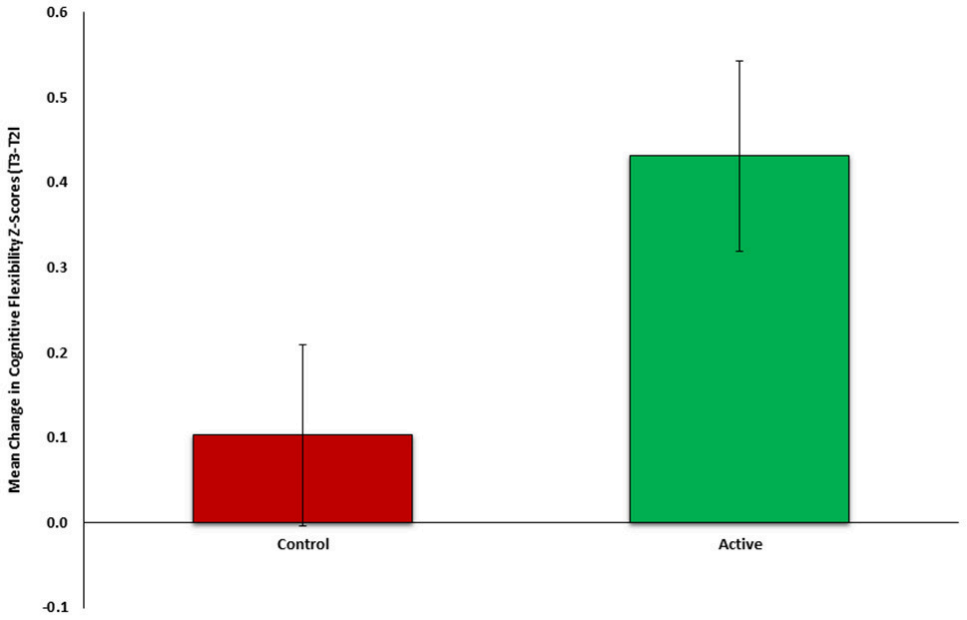
Mean change in cognitive flexibility after intervention (T2-Tl) between active (*n* = 26, green) and control (*n* = 27, red) groups of Study 2.

To test the first part of our auxiliary hypothesis, that an early neurocognitive intervention will improve cognitive functioning and reduce PTSD symptoms (Study 2), the mean change in cognitive flexibility after treatment (T2-T1) was compared between the active (*n* = 26) and control group (*n* = 27). In line with our hypothesis, flexibility change was significantly different between groups [*t*_(51)_ = 2.118, *p* = 0.0195], indicating more improvement among the active (M = 0.4310, *SD* = 0.5737) compared to control group (M = 0.1028, *SD* = 0.5546) (see Figure 2). Cohen's effect size value (d = 0.58) represented a moderate to high practical significance.

**Figure 3:**
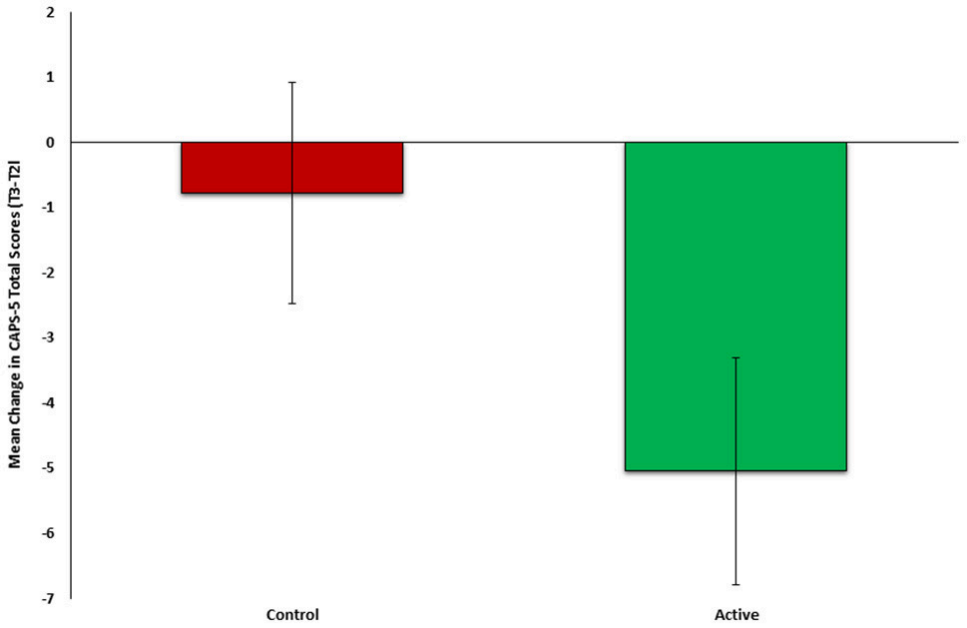
Mean change in PTSD symptom severity (CAPS-5, T3-T2) between active (*n* = 23, green) and control (*n* = 26, red) groups of Study 2.

To test the second part our auxiliary hypothesis, that an early neurocognitive intervention will reduce PTSD symptoms, the mean change in PTSD symptom severity (CAPS-5, T3-T2) was compared between the active (*n* = 23) and control group (*n* = 26). In line with our hypothesis, symptom change was significantly different between groups *(t(47)* = −*2.181, p* = *0.0171)*, indicating more improvement among the active (M = −5.0435, *SD* = 6.3923) compared to control group (M = −0.2692, *SD* = 8.600) (see Figure 3). Cohen's effect size value (d = 0.63) represented a moderate to high practical significance.

**Figure 4:**
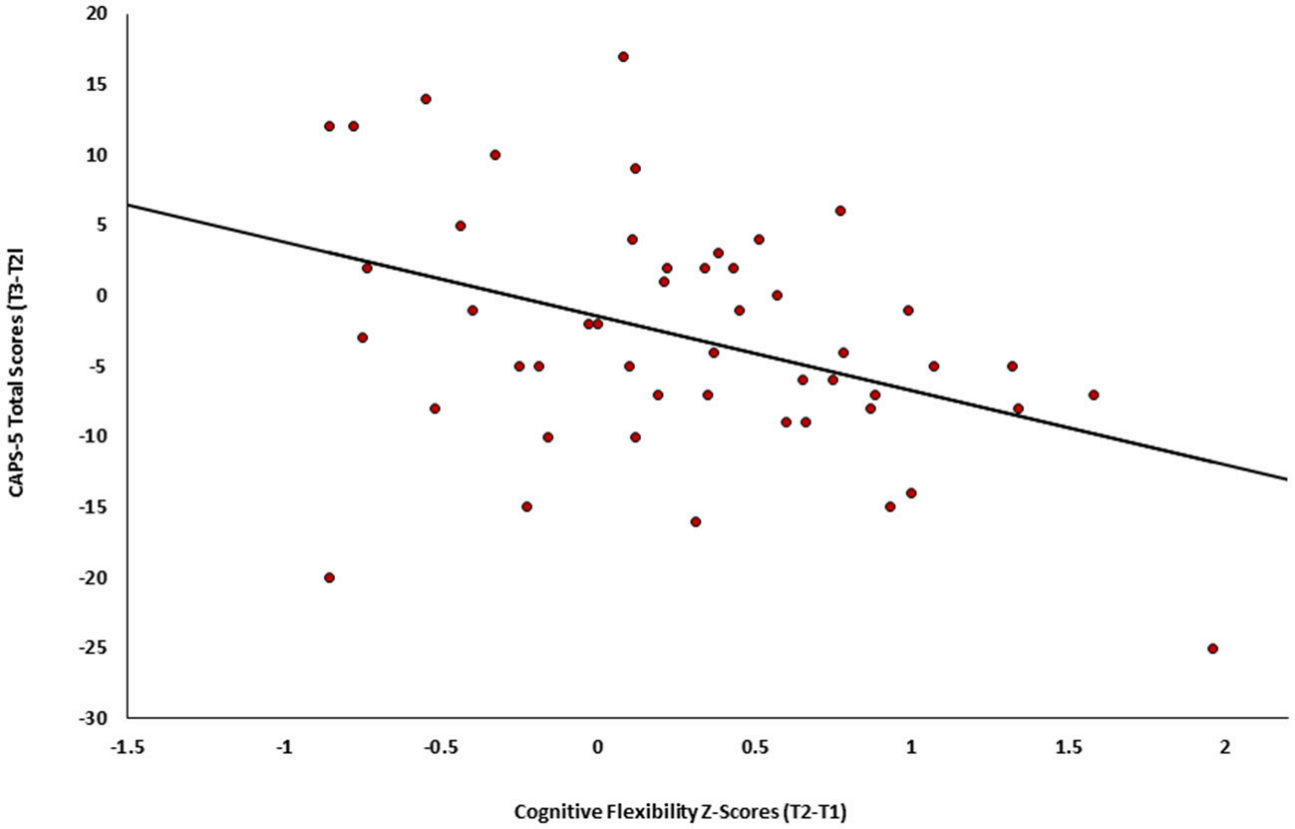
Change in PTSD symptoms severity (CAPS-5, T3-T2) as a function of the change in cognitive flexibility (T2-Tl) among individuals in both active and control groups of Study 2 (*n* = 49).

Finally, the association between change in cognitive flexibility (T2-T1) and subsequent change in PTSD symptom severity (T3-T2) was tested among individuals in both active and control groups (*n* = 49). Results revealed a significant negative correlation *(r* = −0.401*, p* = 0.002), such that individuals who showed greater improvement in cognitive flexibility after treatment (T2-T1) also presented subsequent greater clinical improvement (T3-T2) (see Figure 4).

## Discussion

The current research established a link between cognitive flexibility and PTSD symptom severity among a population of recent trauma survivors, in two independent samples. Consistent with our main hypothesis, we demonstrated that better cognitive flexibility 1 month post-trauma predicted less severe PTSD symptoms at 6 and 14 months post-trauma. It appears that high cognitive flexibility serves as an early resilience factor for PTSD symptom development, whereas low flexibility appears to be a risk factor. This linkage is in line with previous literature linking poor general neurocognitive functions, specifically cognitive flexibility, with increased PTSD symptoms (13, 19). Notably, prior research mostly used single samples and reported small effect sizes (39), whereas our two-independent samples design consisted of large populations of acute PTSD individuals and found medium to large effect sizes.

Consistent with our auxiliary hypothesis, early neurocognitive intervention both improved cognitive functioning and reduced PTSD symptoms. Furthermore, a significant relationship was found between change in early cognitive flexibility and change in subsequent PTSD symptom severity. That is, individuals who exhibit larger improvement in cognitive flexibility measured immediately after treatment, were more likely to show greater clinical improvement later on, and vice versa. These findings potentially suggest that cognitive flexibility serves as a modifiable target preceding and underlying PTSD symptom change. Uncovering such neurocognitive targets may lead to development of mechanism-based interventions specific for PTSD. Although there has been accumulated knowledge regarding cognitive deficits in PTSD, few early neurocognitive based-intervention studies have been conducted (13, 38). However, neurocognitive remediation targeted specifically at aspects of prefrontal function have garnered increasing attention among other psychopathologies, such as depression (40) and schizophrenia (41), reinforcing the vast potential of such interventions. Taken together, our findings draw upon the potential of neuroplasticity-based early interventions which could promote recovery from post-traumatic stress symptoms.

Our findings highlight the significance of cognitive flexibility compared to other neurocognitive functions. The concept of cognitive flexibility is complex, involving several neurocognitive processes including attention, task switching, executive functions and inhibition. In general, it is defined as the readiness with which a person's concept system changes selectively in response to appropriate environmental stimuli. The greater an individual's flexibility, the greater is the likelihood that he will expand and change his categorization and tendency to gain information (19, 39). Furthermore, high cognitive flexibility enables the individual to better differentiate between threat-related and neutral situations, hence to be more flexible and adaptive to changes in the environment. Finally, flexibility assists in the extinction of fear-motivated learning, a core-element in PTSD recovery (39). In general, neuropsychological profiles remain inherently challenging due to the strong dependency between different neurocognitive functions (42). Thus, a deficit or improvement in one neurocognitive structure might be related to several other structures. Nonetheless, it is crucial to try and differentiate these inter-related constructs to target specific mechanisms of the disorder.

Our study implements an integrative and unique prospective approach to the relationship between acute PTSD symptoms and neuropsychological processes. This study was carried out in two large independent samples, at different recruitment sites, administered at different time periods, with different research teams. Nevertheless, we demonstrated similar results in both samples that did not receive treatment, increasing the validity, reliability and generalizability our findings. This study emphasizes the importance of cognitive flexibility both in spontaneous recovery and in targeted neurocognitive interventions. For PTSD.

Although our findings are promising, this work has several limitations. First, only one task with several subscales was used to assess cognitive flexibility. Additional measures and methods, as well as in-person and more thorough assessment, could provide additional insights into the complexity of cognitive flexibility, and neuropsychological functioning in general. Second, the majority of our participants suffered from a single trauma, mostly motor vehicle accidents (MVAs). Future work may explore the relationship between cognitive flexibility and PTSD symptom severity among varying traumatic events, such as terror attacks, interpersonal violence, and continuous traumatic experiences. Nevertheless, our results suggest that this intervention may be effective in treating MVA trauma survivors. Third, it is important to note that we cannot determine whether low cognitive flexibility serves as a pre-existing vulnerability factor, a result of the trauma, or an interaction between these two. Future research should add measurement of neurocognitive functions before trauma, in order to determine which option is the most plausible. Finally, this study did not assess early life trauma, thus limiting our ability to differentiate any earlier impact of trauma on neurocognitive impairment. However, the study excluded participants with chronic PTSD and other major affective disorders thus reducing effect of previous neurocognitive dysfunction due to psychopathology.

In summary, our findings shed light on the underlying neurocognitive mechanisms of PTSD symptoms, and demonstrate the effectiveness of an early neurocognitive intervention in relieving PTSD symptoms. Such findings may guide early mechanism-driven, stage-specific interventions for PTSD, thus improving life quality of trauma survivors and increasing cost-effectiveness of personalized interventions.

## Availability of data and materials

Data currently compiled and QA'd for analyses. Will be available upon demand once brought to maturity by contacting the study PI.

## Ethics statement

The research study meets all ethical regulations as required by ethics committee in Shaare-Zedek Medical Center (Reference number 0018/14) and in Tel-Aviv Sourasky Medical Center (Reference number 0207/14). All subjects gave written informed consent in accordance with the Declaration of Helsinki. Study 2 ClinicalTrials.gov registration number: NCT02085512.

## Author contributions

NF, ZB, and NK carried out the procedural aspects of the study. NF, ZB and NK carried out the research assistants training, guidance and monitoring, management of participants and QA of data. NG, MH, and MA have conducted clinical and neurocognitive assessments. HS assisted in establishing and maintaining the connection with the ER at TLV site. OM and PH managed the hospital interfaces specifically with the ER of the medical centers. NF and ZB drafted the manuscript and finalized it. GF contributed to the study design and statistical analysis. RA, TH, AS, AE, and IL initiated and supervised all procedures at TLV site. AS designed, obtained funding and oversaw the implementation of study 2. AS TH and IL designed, obtained funding and oversaw the implementation of Study 1. AS and AE initiated and supervised all procedures at SZ site. All authors have read and approved the final manuscript.

### Conflict of interest statement

The authors declare that the research was conducted in the absence of any commercial or financial relationships that could be construed as a potential conflict of interest.

## References

[1] Kessler RC Sonnega A Bromet E Hughes M Nelson CB. Posttraumatic stress disorder in the National Comorbidity Survey. Arch Gen Psychiatry (1995) 52:1048–60. 10.1128/AAC.03728-14 7492257

[2] Kessler RC. Posttraumatic stress disorder: the burden to the individual and to society. J Clin Psychiatry (2000) 61(Suppl. 5):4–12; discussion 13–4. 10.1002/(ISSN)1097-4679 10761674

[3] Galatzer-Levy IR Ankri Y Freedman S Israeli-Shalev Y Roitman P Gilad M. Early PTSD symptom trajectories: persistence, recovery, and response to treatment: results from the jerusalem trauma outreach and prevention study (J-TOPS). PLoS ONE (2013) 8:e70084. 10.1371/journal.pone.0070084 23990895 PMC3750016

[4] Koren D Arnon I Klein E. Long term course of chronic posttraumatic stress disorder in traffic accident victims: a three-year prospective follow-up study. Behav Res Ther. (2001) 39:1449–58. 10.1016/S0005-7967(01)00025-0 11758702

[5] Perkonigg A Pfister H Stein MB Höfler M Lieb R Maercker A. longitudinal course of posttraumatic stress disorder and posttraumatic stress disorder symptoms in a community sample of adolescents and young adults. Am J Psychiatry (2005) 162:1320–7. 10.1176/appi.ajp.162.7.1320 15994715

[6] Galatzer-Levy IR Karstoft KI Statnikov A Shalev AY. Quantitative forecasting of PTSD from early trauma responses: a machine learning application. J Psychiatr Res. (2014) 59:68–76. 10.1016/j.jpsychires.2014.08.017 25260752 PMC4252741

[7] Stein DJ Karam EG Shahly V Hill ED King A Petukhova M. Post-traumatic stress disorder associated with life-threatening motor vehicle collisions in the WHO World Mental Health Surveys. BMC Psychiatry (2016) 16:257. 10.1186/s12888-016-0957-8 27449995 PMC4957291

[8] Galatzer-Levy IR Bonanno GA Bush DEA LeDoux JE. Heterogeneity in threat extinction learning: substantive and methodological considerations for identifying individual difference in response to stress. Front Behav Neurosci. (2013) 7:55. 10.3389/fnbeh.2013.00055 23754992 PMC3665921

[9] Ursano RJ Fullerton CS Epstein RS Crowley B Kao TC Vance K. Acute and chronic posttraumatic stress disorder in motor vehicle accident victims. Am J Psychiatry (1999) 156:589–95. 10.1176/ajp.156.4.589 10200739

[10] Shalev AY Ankri Y Israeli-Shalev Y Peleg T Adessky R Freedman S. Prevention of posttraumatic stress disorder by early treatment. Arch Gen Psychiatry (2012) 69:166–76. 10.1001/archgenpsychiatry.2011.127 21969418

[11] Shalev AY Ankri YLE Peleg T Israeli-Shalev Y Freedman S. Barriers to receiving early care for PTSD: results from the jerusalem trauma outreach and prevention study. Psychiatric Serv. (2011) 62:765–73. 10.1176/ps.62.7.pss6207_0765 21724790

[12] Shalev A Liberzon I Marmar C. Post-traumatic stress disorder. New Engl J Med. (2017) 376:2459–69. 10.1056/NEJMra1612499 28636846

[13] Scott JC Matt GE Wrocklage KM Crnich C Jordan J Southwick SM. A quantitative meta-analysis of neurocognitive functioning in posttraumatic stress disorder. Psychol Bull. (2015) 141:105–40. 10.1037/a0038039 25365762 PMC4293317

[14] Johnsen GE Asbjørnsen AE. Consistent impaired verbal memory in PTSD: a meta-analysis. J Affect Disord. (2008) 111:74–82. 10.1016/j.jad.2008.02.007 18377999

[15] Samuelson KW. Post-traumatic stress disorder and declarative memory functioning: A review. Dialog Clin Neurosci. (2011) 13:346–51. 10.1016/j.tics.2006.04.007 22033732 PMC3182004

[16] Aupperle RL Melrose AJ Paulus MP. Executive function and PTSD: disengaging from trauma. Neuropharmacology (2012) 62:686–94. 10.1016/J.NEUROPHARM.2011.02.008 21349277 PMC4719148

[17] Polak AR Witteveen AB Reitsma JB Olff M. The role of executive function in posttraumatic stress disorder: a systematic review. J Affect Dis. (2012) 141:11–21. 10.1016/j.jad.2012.01.001 22310036

[18] Casada JH Roache JD. Behavioral inhibition and activation in posttraumatic stress disorder. J Nerv Mental Dis. (2005) 193:102–9. 10.1097/01.nmd.0000152809.20938.37 15684912

[19] Hart RP Bagrodia R Rahman N Bryant RA Titcombe-Parekh R Marmar CR. Neuropsychological Predictors of Trauma Centrality in OIF/OEF Veterans. Front Psychol. (2017) 8:1120. 10.3389/fpsyg.2017.01120 28713319 PMC5492846

[20] Koenen KC Driver KL Oscar-Berman M Wolfe J Folsom S Huang MT. Measures of prefrontal system dysfunction in posttraumatic stress disorder. Brain Cogn. (2001) 45:64–78. 10.1006/BRCG.2000.1256 11161363

[21] Leskin LP White PM. Attentional networks reveal executive function deficits in posttraumatic stress disorder. Neuropsychology (2007) 21:275–84. 10.1037/0894-4105.21.3.275 17484590

[22] Vasterling JJ Constans JI Brailey K Sutker PB. Attention and memory dysfunction in posttraumatic stress disorder. Neuropsychology (1998) 12:125–33. 10.1037/0894-4105.12.1.125 9460740

[23] Pineles SL Shipherd JC Mostoufi SM Abramovitz SM Yovel I. Attentional biases in PTSD: More evidence for interference. Behav Res Ther. (2009) 47:1050–7. 10.1016/J.BRAT.2009.08.001 19716122

[24] Bryant RA Felmingham KL Kemp AH Barton M Peduto AS Rennie C. Neural networks of information processing in posttraumatic stress disorder: a functional magnetic resonance imaging study. Biol Psychiatry (2005) 58:111–8. 10.1016/j.biopsych.2005.03.021 16038681

[25] Falconer E Bryant R Felmingham KL Kemp AH Gordon E Peduto A. The neural networks of inhibitory control in posttraumatic stress disorder. J Psychiatry Neurosci. (2008) 33:413–22. 18787658 PMC2527717

[26] Kaplan Z Weiser M Reichenberg A Rabinowitz J Caspi A Bodner E. Motivation to serve in the military influences vulnerability to future posttraumatic stress disorder. Psychiatry Res. (2002) 109:45–9. 10.1016/S0165-1781(01)00365-1 11850050

[27] Scholz J Klein MC Behrens TEJ Johansen-Berg H. Training induces changes in white-matter architecture. Nat Neurosci. (2009) 12:1370–1. 10.1038/nn.2412 19820707 PMC2770457

[28] Takeuchi H Sekiguchi A Taki Y Yokoyama S Yomogida Y Komuro N. Training of working memory impacts structural connectivity. J Neurosci. (2010) 30:3297–303. 10.1523/JNEUROSCI.4611-09.2010 20203189 PMC6634113

[29] Voss MW Prakash RS Erickson KI Boot WR Basak C Neider MB. Effects of training strategies implemented in a complex videogame on functional connectivity of attentional networks. NeuroImage (2012) 59:138–48. 10.1016/j.neuroimage.2011.03.052 21440644

[30] Bremner JD Elzinga B Schmahl C Vermetten E. Structural and functional plasticity of the human brain in posttraumatic stress disorder. Progress Brain Res. (2008) 167:171–86. 10.1016/S0079-6123(07)67012-5 18037014 PMC3226705

[31] Mahan AL Ressler KJ. Fear conditioning, synaptic plasticity and the amygdala: Implications for posttraumatic stress disorder. Trends Neurosci. (2012) 35:24–35. 10.1016/j.tins.2011.06.007 21798604 PMC3206195

[32] Olesen PJ Westerberg H Klingberg T. Increased prefrontal and parietal activity after training of working memory. Nat Neurosci. (2004) 7:75–9. 10.1038/nn1165 14699419

[33] Weathers FW Bovin MJ Lee DJ Sloan DM Schnurr PP Kaloupek DG Psychological assessment the clinician-administered PTSD scale for DSM−5 (CAPS- 5): development and initial psychometric evaluation in military veterans. Psychol Assess. (2017) 30:383–95. 10.1037/pas0000486 28493729 PMC5805662

[34] First MB Spitzer RL Gibbon M Williams JBW Structured Clinical Interview for DSM-IV Axis I Disorders, Clinician Version (SCID-CV) for DSMIV. New York, NY: Columbia University Press (1997).

[35] Silverstein SM Berten S Olson P Paul R Williams LM Cooper N. Development and validation of a World-Wide-Web-based neurocognitive assessment battery: WebNeuro. Behav Res Methods (2007) 39:940–9. 10.3758/BF03192989 18183911

[36] Stroop JR Stroop color word test. J Exp Physiol. (1935) 643–62. 10.1007/978-0-387-79948-3

[37] Reitan R Validity of the trail making test as an indicator of organic brain damage. Percept Motor Skil. (1958) 8:271–6. 10.2466/PMS.8.7.271-276

[38] Fine NB Achituv M Etkin A Merin O Shalev AY. Evaluating web-based cognitive-affective remediation in recent trauma survivors: study rationale and protocol. Eur J Psychotraumatol. (2018) 9:1442602. 10.1080/20008198.2018.1442602 29535847 PMC5844026

[39] Scott WA Cognitive complexity and cognitive flexibility. Sociometry (1962) 25:405 10.2307/2785779

[40] Siegle GJ Price RB Jones NP Ghinassi F Painter T Thase ME You gotta work at it: Pupillary indices of task focus are prognostic for response to a neurocognitive intervention for rumination in depression. Clin Psychol Sci. (2014) 2:455–71. 10.1177/2167702614536160

[41] Medalia A Saperstein AM. Does cognitive remediation for schizophrenia improve functional outcomes? Curr Opin Psychiatry (2013) 26:151–7. 10.1097/YCO.0b013e32835dcbd4 23318663

[42] Lezak MD Howieson DB Loring DW Hannay HJ Fischer JS. Neuropsychological Assessment. 4th ed. New York, NY: Oxford University Press (2004).

